# Status and analysis of undetected plague cases in Yunnan Province, China

**DOI:** 10.3389/fpubh.2024.1408025

**Published:** 2024-09-04

**Authors:** Chao Su, Biao Duan, Qun Duan, Zhaokai He, Hanyu Sha, Yun Liang, Ennian Pu, Shuai Qin, Ran Duan, Dongyue Lyu, Wenbao Li, Deming Tang, Peng Zhang, Meng Xiao, Lianxu Xia, Huaiqi Jing, Xin Wang, Zihou Gao, Biao Kan

**Affiliations:** ^1^National Institute for Communicable Disease Control and Prevention, Chinese Center for Disease Control and Prevention, Beijing, China; ^2^Plague Center, Yunnan Institute for Endemic Disease Control and Prevention, Dali, China; ^3^Hangzhou Center for Disease Control and Prevention, Hangzhou, China; ^4^Heqing Center for Disease Control and Prevention, Dali, China; ^5^Dongcheng District Center for Disease Control and Prevention, Beijing, China; ^6^Tianjin Center for Disease Control and Prevention, Tianjin, China

**Keywords:** plague, *Yersinia pestis*, *Rattus flavipectus* plague focus, undetected plague cases, IHA

## Abstract

**Background:**

The virulence of *Yersinia pestis* strains in the *Rattus flavipectus* plague focus is relatively low. The purpose of this study was to investigate the undetected, sporadic plague cases in plague foci and provide the basis for plague prevention and control.

**Methods:**

A 3-year-old plague-confirmed case was investigated in the *R. flavipectus* plague focus of Yunnan Province in 2020 due to the intensive screening for fever symptoms during the coronavirus disease 2019 (COVID-19) pandemic. Epidemiological investigation, laboratory testing, and clinical treatment were conducted for the case. The expanded survey was carried out around the case within a 7-km radius, including the resident population, domesticated dogs, and rats. PCR and indirect hemagglutination tests were performed on the collected samples.

**Results:**

The isolation rates of *Y. pestis* were 100.0% (7 out of 7) in dead rats and 4.00% (3 out of 75) in live rats in the survey area of the foci. A total of 5.00% (6 out of 120) of the domesticated dogs were F1 antibody positive. Nine local people were determined for plague infection recently (0.92%, 9 out of 978). The locations of human cases coincided with the *Y. pestis* epidemic area among the animals.

**Conclusion:**

This study discovered the existence of plague cases that had not been detected by routine surveillance in the *R. flavipectus* plague focus, and the actual epidemic of human infection may be underestimated.

## 1 Introduction

Plague has had a devastating impact on humans throughout history (1, 2). With the advent of antimicrobials, especially streptomycin, critical progress has been made in the treatment of plague (3, 4), and the survival rate of bubonic plague has greatly increased. Since 2019, 17 human plague cases have been reported in China (mainly in Inner Mongolia, Ningxia, and Tibet). The current active plague foci in China, where both animal plague are prevalent and human plague cases occur, include the *Marmota* plague foci, *Meriones unguiculatus* plague focus of the Inner Mongolia Autonomous Region, *Rattus flavipectus* plague focus of the Yunnan Province, and *Apodemus chevrieri*–*Eothenomys miletus* plague focus of the highlands of northwestern Yunnan Province (5–8). The house rats are the main host animals in the *Rattus flavipectus* plague focus in Yunnan. The virulence of *Yersinia pestis* strains in the *R. flavipectus* plague focus is much lower than the strains in *Marmota* plague foci or *Meriones unguiculatus* plague foci, resulting in the main bubonic plague cases but rare pneumonic plague cases and low case fatality rate (8, 9). As antibiotic abuse has become a serious problem, most fever cases were treated with broad-spectrum antibiotics without clinical tests (10, 11). Some of the human plague cases in the *Rattus flavipectus* plague focus might be cured by broad-spectrum antibiotic treatment without etiological detection. Given this, it may be highly likely that undetected, sporadic human plague cases and even local outbreaks occur in the foci. Studies have found that undetected cases have occurred in other parts of China (12, 13).

The monitor for the animal plague in China mainly relied on active surveillance (serological and etiological detection among the animals or fleas) in the epidemic foci, while the monitor for the human plague mainly relied on passive surveillance (case reports). Human plague cases could easily be misdiagnosed as common infectious diseases and treated incorrectly in primary hospitals. For example, two human plague cases occurred in the *Meriones unguiculatus* plague focus in the Inner Mongolia Autonomous Region, China, at the end of 2019 (6, 14). The patient was initially treated for pneumonia, not plague, which led to a delay of approximately 2 weeks before a definitive diagnosis when two cases were sent to Beijing for medical treatment.

The intensive screening of fever and respiratory symptoms was implemented during the COVID-19 pandemic in 2020, which greatly improved the unexpected detection of human plague cases in Yunnan Province.

## 2 Materials and methods

### 2.1 Investigation of the plague-confirmed case in Yunnan Province, 2020

A human plague-confirmed case was diagnosed during the intensive screening of fever symptoms to COVID-19 response in Menghai County, Yunnan Province, 2020. Epidemiological investigation, laboratory testing, and clinical treatment were conducted for the case. Plague host animals in the area were investigated according to the range of movement of the patient in the 10 days before the onset of illness. The host animals and flea vectors that the patient may have contacted within 10 days before the onset should be investigated. Lymph node puncture fluid and blood of the patient were collected to detect *Y. pestis-*specific genes, F1 antigen, and isolate *Y. pestis* strains. The serum of the patient was detected with the F1 antibody.

### 2.2 The human case related plague epidemic survey

#### 2.2.1 Survey area

**Figure 1:**
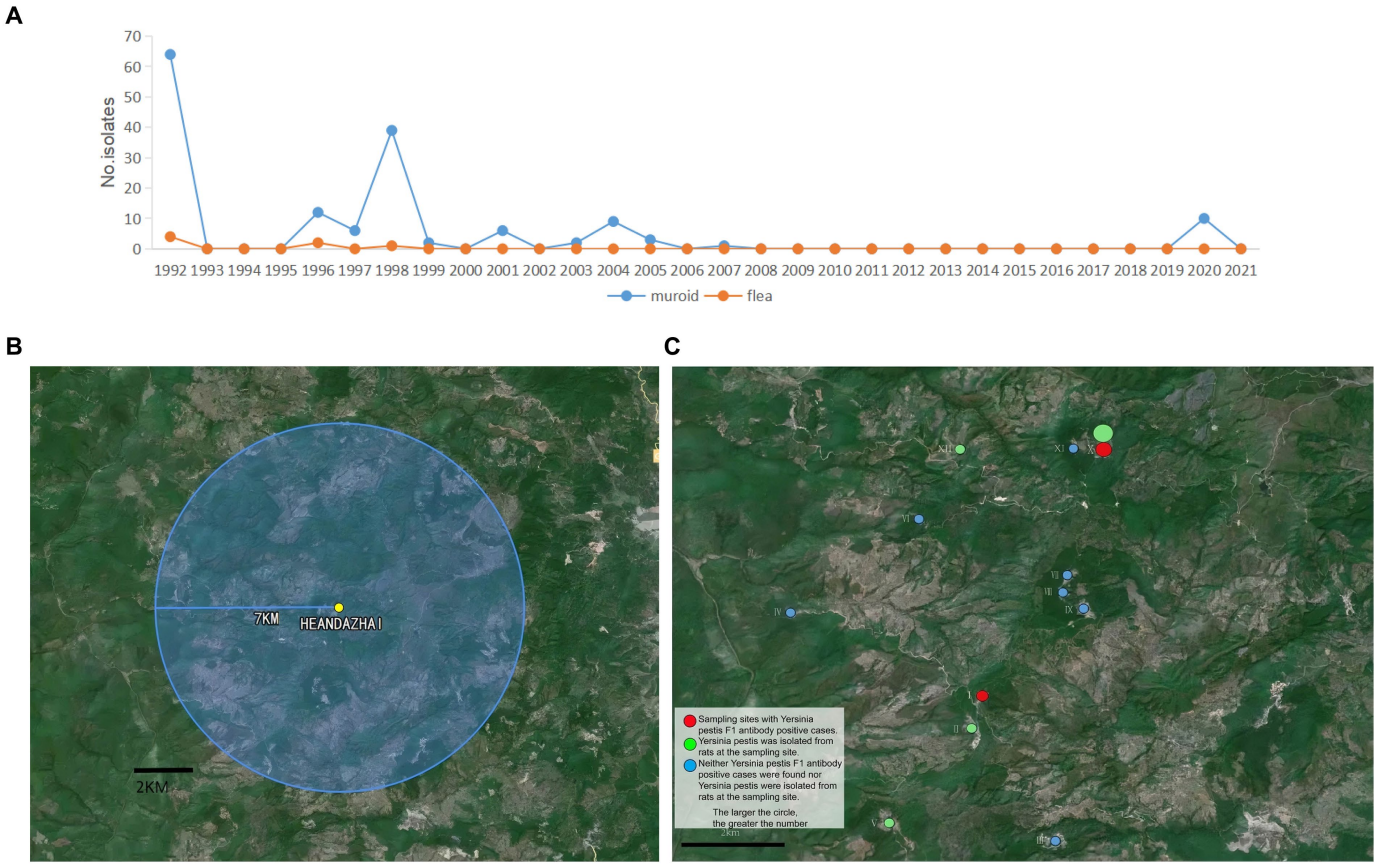
Historical and 2020 plague prevalences in Menghai County. **(A)** The number of *Yersinia pestis* strains isolated in Menghai County in 1992–2021. **(B)** A 7 km survey area around He’an Dazhai. **(C)** Green circles indicate that there were *Y. pestis* F1 antibody-positive cases at the sample sites (larger circles indicate more cases); red circles indicate that *Y. pestis* strains were isolated from rats at the sampling sites (larger circles indicate more strains); and blue circles indicate that no *Y. pestis* F1 antibody-positive cases or *Y. pestis* strains in rats were found at the sampling sites.

Taking the location of the confirmed case (He’an Dazhai) as the epidemiological central point, 12 investigation sites were set up within a 7 km radius (15). These 12 sites included Lao Dong (I), Nan Mo (II), Jiu Hai (III), Man Bang (IV), Man Ma (V), Nan Sheng (VI), Jiuguo Dazhai (VII), Jiuguo Xiaozhai (VIII), Jiuguo Xinzhai (IX), He’an Dazhai (X), He’an Xiaozhai (XI), and Bang He (XII) (Figure 1).

#### 2.2.2 Sample collection and *Yersinia pestis* detection

This investigation took place among the resident population, domesticated dogs, and rats in the survey area. A total of 978 humans and 120 domesticated dogs were involved and the blood samples were collected by doctors and veterinarians to detect the F1 antibody. Furthermore, 75 live rats were captured and seven dead rats were found during the survey to isolate the *Y. pestis* strains. These animals were collected in these 12 investigation sites (Table 1). Live rats were caught by placing traps in the survey area, dead rats were self-killed rats that appeared in the survey area, and the blood samples of domesticated dogs were collected as much as possible as long as domesticated dogs were found in the survey area. The samples were collected with people’s informed consent.

**Table 1:** Samples collected at 12 sites.

Sites	Humans		Domesticated dogs		Live rats		Dead rats	
	No. of serum	No. of F1 antibody positive	No. of serum	No. of F1 antibody positive	No. of rats	No. of strains	No. of rats	No. of strains
Lao Dong I	145	6	16	2	8	1	2	2
Nan Mo II	103	1	10	1	0	0	0	0
Jiu Hai III	129	0	15	0	0	0	0	0
Man Bang IV	107	0	17	1	0	0	0	0
Man Ma V	120	1	3	0	0	0	0	0
Nan Sheng VI	70	0	14	0	2	0	0	0
Jiuguo Dazhai VII	89	0	9	0	0	0	0	0
Jiuguo Xiaozhai VIII	56	0	4	0	16	0	0	0
Jiuguo Xinzhai IX	24	0	2	0	4	0	0	0
He’an Dazhai X	80	0	18	2	28	2	5	5
He’an Xiaozhai XI	36	0	6	0	3	0	0	0
Bang He XII	19	1	6	0	1	0	0	0
Non-sampling points	-	-	-	-	13	0	-	-
Total	978	9	120	6	75	3	7	7

“-” indicates that no data is collected.

### 2.3 Laboratory testing

The DNA of lymph node puncture fluid and blood were extracted, respectively (Blood & Tissue Kit, Qiagen 69506, Hilden, Germany), followed by PCR detection of *Y. pestis-*specific genes (*caf1* and *pla*) (16). The primer sequences were as follows:

*caf1*-F: 5′-GGAACCACTAGCACATCTGTT-3′
*caf1*-R: 5′-ACCTGCTGCAAGTTTACCGCC-3′
*pla*-F: 5′-ACTACGACTGGATGAATGAAAATC-3′
*pla*-R: 5′-GTGACATAATATCCAGCGTTAATT-3′

Reverse indirect hemagglutination assay (RIHA, Qinghai Province Endemic Disease Prevention and Control Institute, Xining, Qinghai Province, China) was used to detect plague F1 antigen in the lymph puncture fluid and plasma (16–19).

Indirect hemagglutination assay (IHA, Qinghai Province Endemic Disease Prevention and Control Institute, Xining, Qinghai Province, China) was used to detect plague F1 antibody in serum (antibody titer ≥1:16 indicated a positive result) (16, 17, 19). The prevalence of plague in humans, domesticated dogs, and live rats captured around the human confirmed case was investigated.

To isolate and identify *Y. pestis*, the spleen or the bone marrow of rats (live rats and self-dead rats) were inoculated on *Yersinia* selective medium (CIN medium) at 28°C for 48 h. Bacterial colonies were identified by *Y. pestis* bacteriophage (*Yersinia pestis* diagnostic phage kit, Lanzhou Institute of Biological Products Co., Ltd.). *Yersinia pestis* phage was dropped to the suspected bacteria and observed for lysis phenomena. If there is a bacteriophage zone and there is no growth of bacteria in the zone, it can be identified as *Y. pestis* or through PCR detection for *caf1* and *pla* genes (20, 21). At the same time, nucleic acid was extracted from the heart, liver, spleen, lung, and kidney tissues of live rats and self-dead rats after grinding the tissues for PCR detection for *pla* and *caf1* genes.

### 2.4 Historical epidemiological data

Historical epidemiological background data on plague from 1992 to 2021 in Menghai County, where the confirmed case is located, were obtained from the routine surveillance data of the Yunnan Institute for Endemic Disease Control and Prevention, China.

## 3 Results

### 3.1 Historical epidemiological background of plague in Menghai County

Menghai County, Yunnan Province, is one of the areas where *R. flavipectus* human plague cases are highly prevalent. In the past, especially in the 55 years from 1886 to 1940, 25 years (1886, 1891, 1899, 1900, 1904 to 1907, 1909, 1913, 1914, 1917, 1919, 1921, 1923, 1925, 1927, 1932–1934, and 1936–1940) of plague epidemics with varying degrees were recorded. After 51 years of plague occurring in a low-level state, plague reoccurred in Menghai County, which was identified as the natural plague focus county in February 1992. In total, 11 cases of bubonic plague were reported, and 64 *Y. pestis* strains were isolated from the rats and four strains from the fleas in Menghai County in 1992. *Yersinia pestis* was isolated in subsequent years: 1996, 1997, 1998, 1999, 2001, 2003, 2004, 2005, 2007, and 2020 (Figure 1A). In 1998, the plague epidemic among animals was the most severe, with 24 epidemic sites, indicating a strong spread of plague in that year. In 1992, the plague also spread to humans, but no human cases have been reported since then. After 2007, the plague among animals was in a low-level state. From 2008 to 2019, *Y. pestis* was not isolated from either the host animal rat or the vector flea (Figure 1A). By 2020, *Y. pestis* was isolated from the rats.

### 3.2 Investigation of plague-confirmed case in Yunnan Province, 2020

A 3-year-old boy was diagnosed with the plague-confirmed case in 2020 and was from He’an Dazhai, Xiding Township, Menghai County, Yunnan Province. On 20 September 2020, the boy had a fever for unknown reasons (up to 40.2°C). Fever screening had been intensive during the COVID-19 pandemic, and the child’s symptoms were taken seriously. As accompanied by left inguinal lymph node enlargement and obvious tenderness, the boy was preliminarily identified as a suspected case of bubonic plague. The patient was admitted to the hospital and received treatment on 21 September 2020.

The patient’s blood and lymph node puncture fluid were collected for laboratory testing on 21 September. PCR showed that the lymph node puncture fluid was *caf1* and *pla* positive while the blood test was negative. RIHA results were negative. *Yersinia pestis* was not isolated from the patient. The F1 antibody in the serum was negative during that time. The patient’s blood was collected again on 26th September, 27th September, and 6th October, and F1 antibodies tested positive with an initial antibody titer of 1: 64, 1:128, and 1:128, respectively.

The epidemiological investigation also found that there were dead rats in the patient’s and neighbors’ houses within 10 days before the plague-confirmed case onset. The boy patient had the habit of climbing and sitting on the ground. The patient’s family house was a traditional Dai-style wooden building. It is a nearly square building with two floors. People live on the upper level, and livestock are kept on the lower level. This building is suitable for the growth and reproduction of the main host and the main vector of plague, and the rats could crawl into the house along the wood.

**Figure 2:**
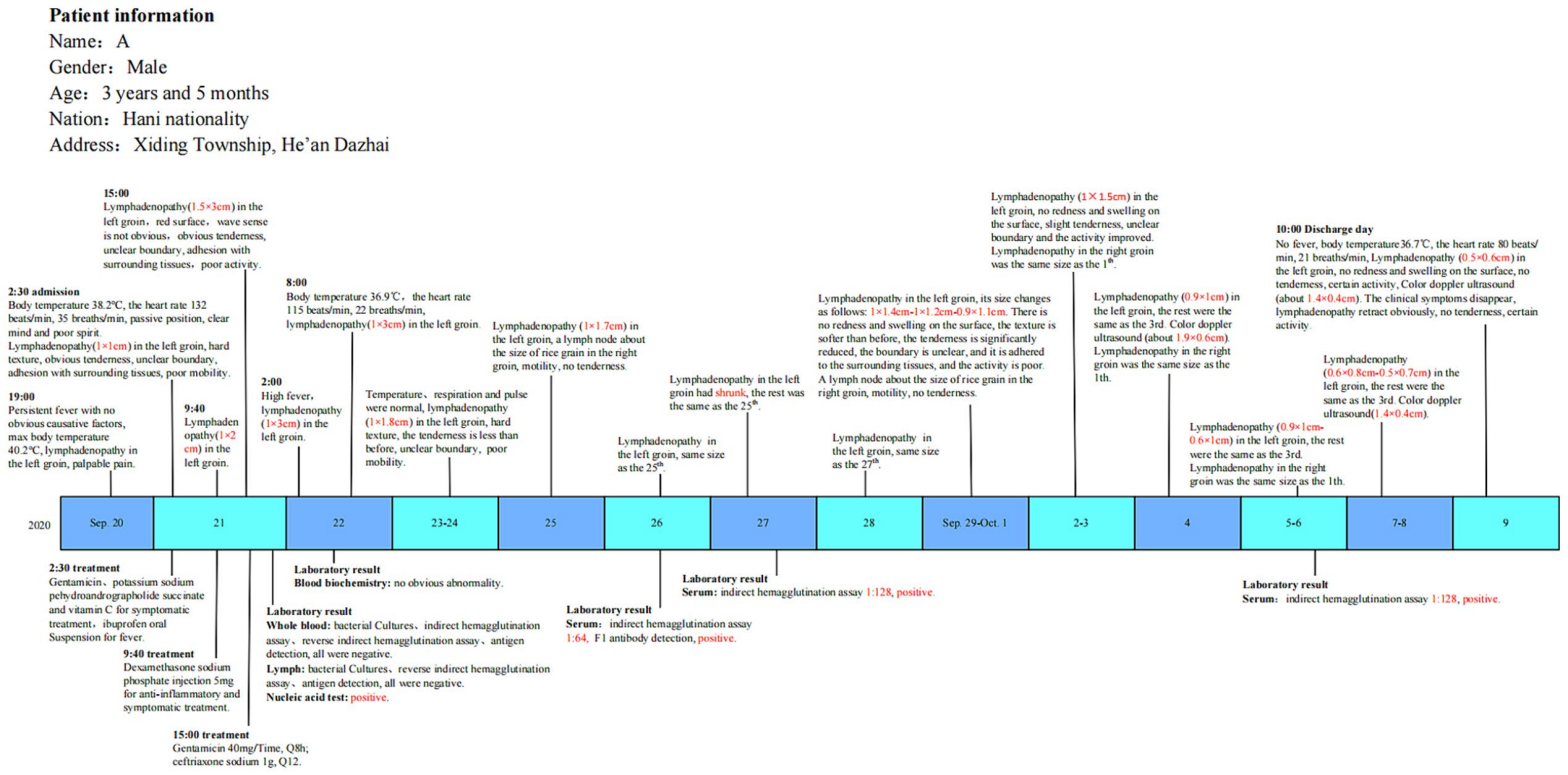
Investigation timeline of the patient.

Based on the clinical manifestations, epidemiological findings, and laboratory tests, the case was identified as a bubonic plague-confirmed case (Figure 2).

### 3.3 Investigation of the plague epidemic among local residents

Screening was conducted at 12 sites in Xiding Township, Menghai County, Yunnan Province, within 7 km of He’an Dazhai (epidemiological center) (Figure 1B).

Indirect hemagglutination assay was performed on the sera of 978 residents (Table 1). Nine human serum samples (0.92%, 9/978) were tested positive for F1 antibody. Among the F1 antibody-positive human serum samples, six people in Laodong (I) were tested positive. One person had a titer of 1:64, four persons had a titer of 1:128, and one person had a titer of 1:256. The titers were 1:32 in Nan Mo (II), 1:128 in Man Ma (V), and 1:64 in Bang He (XII), respectively. There is no plague vaccination all over China at present, and we could regard the nine F1 antibody-positive residents as undetected plague cases.

### 3.4 Investigation of plague epidemic among animals

The sera of 120 domesticated dogs were collected and six (5.00%, 6 out of 120) domesticated dogs were tested positive (Table 1). Among them, the titers were 1:16 to 1:128 in Lao Dong (I), 1:32 to 1:64 in He’an Dazhai (X), 1:32 in Nan Mo (II), and 1:16 in Man Bang (IV).

A total of 75 live rats were captured and seven dead rats were found in the survey area (Table 1). A total of 10 *Y. pestis* strains were isolated, seven (100.00%, 7 out of 75) from dead rats, and three (4.00%, 3 out of 75) from live rats. These comprised seven (five from dead rats and two from live rats) from He’an Dazhai (X) and three (two from dead rats and one from a live rat) from Lao Dong (I) (Figure 1C).

## 4 Discussion

The plague foci in Yunnan Province, China, were age-long (22). Before 1950, Yunnan Province experienced three plague epidemic periods (1772–1855, 1856–1937, and 1938–1949). During the two initial high-incidence periods, millions of people died, but the scale of the epidemic gradually decreased over time. It was recorded that approximately 253,000 people died of plague during 1772–1855, approximately 733,500 during 1856–1937, and 4,804 during 1938–1949 (with a case fatality rate of 46.5% among the 10,450 plague cases in the third plague pandemic period) (9). Due to a lack of etiological detection before 1950, those plague cases were mainly determined based on the close association with the massive rats deaths in residential areas. The *R. flavipectus* plague focus of Yunnan Province existed in the 18th century (23). *Yersinia pestis* strains in the *R. flavipectus* plague focus had relatively weak virulence compared to strains in other plague foci (24). The epidemic form is similar to that of the Madagascar plague (25–27).

From 1950 to 1954, the scale of the plague epidemic in Yunnan Province gradually decreased, and it almost disappeared from 1955 to 1989. From 1990 to 2005, the plague epidemic increased again, and the reported cases faded away after 2005 (8, 9), posing a certain threat to the health of residents. The imperfect epidemic surveillance and reporting system, the lag in case reporting, and the lack of clinical detection methods in local hospitals may be the main reasons for not being able to detect plague cases on time in the past. Due to some circumstances (inconvenient medical treatment, blocked information, patients who take antibiotics by themselves and fail to take the initiative to seek medical treatment, etc.), the case reports are less than the actual situation, and the cases cannot be detected, diagnosed, and treated on time. The key to solving these problems is to strengthen animal plague surveillance and provide relevant training for doctors in the affected areas. In addition, fully understanding and improving the defects in the epidemic surveillance and reporting system will increase detection sensitivity and timeliness during potential epidemics. It is very important to monitor the plague among animals in the epidemic focus. Once the plague is found to be prevalent among animals in a certain area, health education will be conducted for local residents, knowledge of plague prevention and control will be publicized, and residents will be allowed to report themselves according to symptoms. At the same time, the doctors in the fever clinic of the local hospital must be kept informed, indicating that the plague is circulating among animals in the area, and residents and local hospitals need to pay attention to the report of suspected plague cases.

A 3-year-old boy was identified from the intensive fever screening in response to the COVID-19 pandemic on 20 September 2020. The boy’s COVID-19 test was negative. There was a high suspicion of plague as the clinical manifestations included inguinal lymph node enlargement and as the location (He’an Dazhai) was a natural plague focus, and then he was confirmed of plague by running the laboratory tests. A survey of the plague epidemic situation in animals and humans within a 7 km radius of the patient was carried out. The results showed an animal plague epidemic in He’an Dazhai and the surrounding areas. *Yersinia pestis* strains were isolated from the rats at two sites, and F1 antibody-positive domesticated dogs were identified at four sites. F1 antibody-positive humans were also identified at four sites. The location of the confirmed case and F1 antibody-positive residents coincided with the *Y. pestis* epidemic area of both rats and domesticated dogs. In this study, *Yersinia pestis* was not isolated from infected humans but only from infected animals, thus the molecular analysis of both was not conducted. Nevertheless, the consistent distribution pattern at all 12 investigation sites within a 7 km radius strongly indicates the threat of animal-to-human plague transmission and is not a coincidence. The nine F1 antibody-positive residents could be determined to be undetected plague cases, as there is no plague vaccination in China at present. Thus, the survey suggested that there was an epidemic of animal plague and multiple cases of human plague.

The majority of the historically detected strains in Yunnan belonged to weak virulence strains (9, 28). Human infection with plague rarely develops into pneumonic plague in the *R. flavipectus* plague focus. The majority of the cases in this plague focus involve bubonic plague, with a long incubation period; the prognosis is usually good with early treatment, with a low mortality rate (8, 9). The infection spreads via flea bites, with few cases of human-to-human transmission. The undetected cases may infect the plague by contacting the infected rats or being bitten by the infected flea. They may have milder symptoms and easily be misdiagnosed as having a cold or acute lymphadenitis. The undetected cases were fortunately cured by the application of broad-spectrum antibiotics such as fluoroquinolones or tetracyclines.

The causes of these plague outbreaks involve many factors, including deficiencies in surveillance systems, case reporting, and epidemic prevention and control. Early outbreak detection and intervention are difficult, especially when the surveillance system is deficient, as plague cases are easily overlooked or misdiagnosed during routine clinical examinations. It is vital to consider multiple possibilities, including various infectious diseases (such as plague), at the screening stage. The routine plague surveillance and diagnostic methods in plague foci should be improved to increase the sensitivity of surveillance and ensure early outbreak detection and intervention.

## 5 Conclusion

The study suggested recognizing that undetected plague cases exist accurately in the active plague foci. We may underestimate the actual epidemic level of human plague cases in local areas.

## Data Availability

The original contributions presented in the study are included in the article/supplementary material, further inquiries can be directed to the corresponding authors.
